# Rate-Adaptive Information Reconciliation for CV-QKD Systems at Low Signal-to-Noise Ratios

**DOI:** 10.3390/e28010010

**Published:** 2025-12-20

**Authors:** Huiting Fu, Jisheng Dai, Yan Feng, Han Hai, Huayong Ge, Peng Huang, Xue-Qin Jiang

**Affiliations:** 1 College of Information and Intelligent Science, Donghua University, Shanghai 201620, China; 2232249@mail.dhu.edu.cn (H.F.); jsdai@dhu.edu.cn (J.D.); hhai@dhu.edu.cn (H.H.); 2 College of Electronic and Information, Shanghai Dianji University, Shanghai 201306, China; fengyan@sdju.edu.cn; 3 State Key Laboratory of Advanced Optical Communication Systems and Networks, Institute for Quantum Sensing and Information Processing, Shanghai Jiao Tong University, Shanghai 200240, China; huang.peng@sjtu.edu.cn

**Keywords:** continuous-variable quantum key distribution, information reconciliation, reconciliation efficiency, secret key rate, log-likelihood ratios

## Abstract

In continuous-variable quantum key distribution (CV-QKD) systems, information reconciliation (IR) is a crucial step that significantly affects the secret key rate (SKR). The fixed-rate error-correcting codes used in IR are highly sensitive to changes in the signal-to-noise ratio (SNR) and cannot maintain a high reconciliation efficiency in practical CV-QKD systems. To address this issue, we first propose a rate-adaptive IR protocol, namely Threshold-based IR (TIR), which changes the code rate of low-density parity-check (LDPC) codes by selectively revealing bits with lower reliability and adjusting their log-likelihood ratios (LLRs). Then, we propose a rate-adaptive IR protocol, namely Sorting-based IR (SIR), which not only adjusts the code rate according to variations in SNR, but also enables the CV-QKD systems to achieve high reconciliation efficiency over a wide range of SNRs. Furthermore, we perform an analysis of the protocols in terms of code rate, reconciliation efficiency, and complexity. The simulation results demonstrate that the proposed protocols outperform other rate-adaptive IR protocols, achieving a reconciliation efficiency higher than 98.5% in the SNR range below −20 dB and maintaining a certain SKR in long-distance transmission.

## 1. Introduction

Quantum key distribution (QKD) [1,2,3] is among the most practical implementations of quantum-information technologies, enabling two spatially separated parties, Alice and Bob, to share random keys in an untrusted environment and theoretically guaranteeing unconditional security [4]. Currently, QKD technology falls into two mainstream types: discrete-variable QKD (DV-QKD) [1,2] and continuous-variable QKD (CV-QKD) [5]. In DV-QKD systems, key information is used to encode the polarization or phase of a single-photon state; in contrast, in CV-QKD systems, the encoding process targets the amplitude and phase quadrature states [6]. CV-QKD systems have attracted considerable research interest due to their compatibility with conventional telecommunication infrastructure [7]. Recent studies have mainly focused on enhancing secure transmission distances and optimizing the secret key rate (SKR) in CV-QKD systems [8,9].

Quantum transmission and post-processing make up a typical CV-QKD system [8]. Bob uses a homodyne or heterodyne detector to test quantum states at random during the quantum transmission [10]. Base sifting [11], parameter estimation [12], information reconciliation (IR) [13,14], and privacy amplification [15] are the four primary processes of post-processing, which is used to obtain secret keys from both parties. Among these, IR is considered the primary technical bottleneck, limiting the transmission distance and the SKR of the system. Ongoing research aims to improve reconciliation efficiency and reduce the frame error rate (FER), thereby improving the SKR [16]. Currently, slice reconciliation [17] and multidimensional reconciliation [18] are currently widely utilized reconciliation techniques.

Considering finite-size effects [12], high-performance error-correcting codes with long block lengths are typically employed in IR to achieve a high reconciliation efficiency. Specifically, multi-edge-type low-density parity-check (MET-LDPC) codes [19,20], as a generalization of LDPC codes [20], have demonstrated near-Shannon-limit performance, which can achieve a high reconciliation efficiency at low signal-to-noise ratios (SNRs). However, fixed-rate LDPC codes cannot maintain a high reconciliation efficiency under fluctuating SNR conditions in practical CV-QKD systems [6,21,22]. Raptor codes [23] and spinal codes [24] with the rateless property can achieve a high reconciliation efficiency over a wider range of SNRs, but require a higher decoding complexity compared to LDPC codes. The rate-adaptive LDPC codes in [16] maintain over 96% reconciliation efficiency by modifying a fixed-size parity-check matrix, though this fixed size is a limitation. The multiple decoding attempt (MDA) protocol was proposed [25], which reveals bits randomly after decoding failure to adjust log-likelihood ratios (LLRs), thereby changing the code rate. However, we follow the definition of bit reliability as proposed in [26], and it has been experimentally demonstrated that bits with lower reliability are significantly more likely to be erroneous, and thus are key contributors to decoding failure.

In this paper, we propose that selectively revealing bits with lower reliability and adjusting their LLRs can change the code rate of LDPC codes. To achieve this, we first introduce a rate-adaptive IR protocol named Threshold-based IR (TIR), where bits with lower reliability below a threshold θ are disclosed. Then, we propose a Sorting-based IR (SIR) protocol, through which bits are sorted by reliability, and the least reliable bits are selectively disclosed. The SIR protocol not only adjusts the code rate according to variations in SNRs, but also achieves high reconciliation efficiency across a wide range of SNRs.

The rest of the paper is structured as follows: In Section 2, we review the fundamentals of IR in brief. In Section 3, we introduce the proposed protocols in detail. In Section 4, we perform an analysis of the proposed protocols. The simulation results of our scheme are shown and analyzed in Section 5. Finally, we conclude this paper in Section 6.

## 2. Preliminaries

IR serves as a crucial procedure in CV-QKD systems, which aims to accomplish error correction while minimizing leakage the information of secret keys, thereby enabling the two communicating parties to acquire symmetric secret keys. IR can be categorized into two main types: direct reconciliation and reverse reconciliation [11]. Reverse reconciliation provides longer transmission distances and maintains a specific SKR during long-distance fiber optic communication at low SNRs, in contrast to direct reconciliation [27].

**Figure 1:**
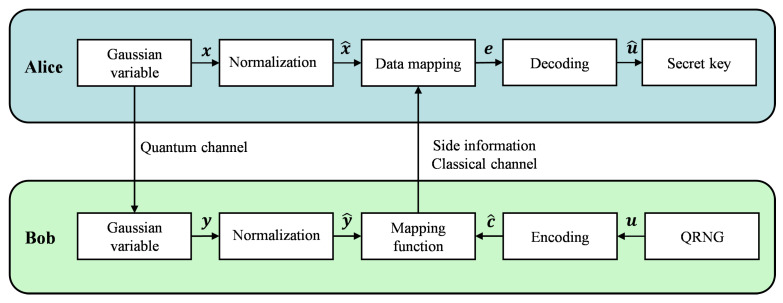
Multidimensional reverse reconciliation schematic diagram.

Multidimensional reverse reconciliation is a popular reconciliation technique for CV-QKD systems due to its superior reconciliation efficiency at low SNRs and low capacity loss. A schematic diagram of CV-QKD systems is presented in Figure 1. The general method of information reconciliation was proposed in [18] and improved in [6,16,28,29]. After going through the data sifting and parameter estimation steps, Alice and Bob obtain correlated quantum sequences x and y; in this context, y=x+z, with z standing for the quantum channel noise. Alice and Bob then split the sequences x and y into *d*-dimensional vectors, and proceed to normalize these vectors respectively.

Using a quantum random number generator (QRNG), Bob creates a secret key consisting of a random binary sequence u. The encoder employs an error-correcting code to encode u into $\hat{c}$, and outputs side information that avoids revealing the secret key. To compute the mapping function $M(\hat{y},\hat{c})$ that acts as part of the side information, we utilize the encoded sequence $\hat{c}$ and the normalized sequence $\hat{y}$. $M(\hat{y},\hat{c})$ is an orthogonal transformation, defined as $M\left(\hat{y},\hat{c}\right)=\sum_{i=1}^{d}\alpha_{i}\left(\hat{y},\hat{c}\right)A_{i}$, where $\alpha_{i}\left(\hat{y},\hat{c}\right)=\left(A_{i}\hat{y}|\hat{c}\right)$; the specific form of matrix $A_{i}$ is available in [18]. Bob then uses a classical channel to send Alice this side information. After receiving $M(\hat{y},\hat{c})$, Alice uses it together with the normalized sequence $\hat{x}$ to obtain e via data mapping. Subsequently, the LDPC code and sum-product algorithm are adopted to recover e into the sequence $\hat{u}$. Once the decoding process succeeds, Alice and Bob will share a symmetrical secret key.

The SKR of a CV-QKD system with one-way multidimensional reverse reconciliation, considering finite-size effects into account, is provided by  [10]

(1) $$K_{\mathrm{finite}}=\frac{n}{N}\left(1-P_{e}\right)\left[\beta I\left(A:B\right)-S_{\epsilon_{\mathrm{PE}}}\left(B:E\right)-\Delta \left(n\right)\right],$$

where *N* is the total amount of data that Alice and Bob exchanged, *n* is the amount of data that was used to extract the keys, and the remaining portion is used for parameter estimation. $P_{e}$ is the reconciliation FER, which is defined as the ratio of discarded secret keys to total secret keys. The reconciliation efficiency is measured by

(2) $$\beta =\frac{R}{C(s)},$$

where *R* denotes the error-correcting code rate and $C\left(s\right)=\frac{1}{2}\log_{2}\left(1+s\right)$ represents the channel capacity when the SNR is set to *s*. I(A:B) represents Alice and Bob’s classical mutual information. The maximum Holevo information that Eve can receive from Bob’s data is $S_{\epsilon_{\mathrm{PE}}}\left(B:E\right)$, where $\epsilon_{\mathrm{PE}}$ is the parameter estimate failure probability [29]. The finite-size offset factor is represented by Δ(n). According to Equation (1), an imperfect reconciliation scheme leads to a reduction in the SKR and imposes limitations on the applicable range of the protocol.

## 3. The Proposed Protocols

**Figure 2:**
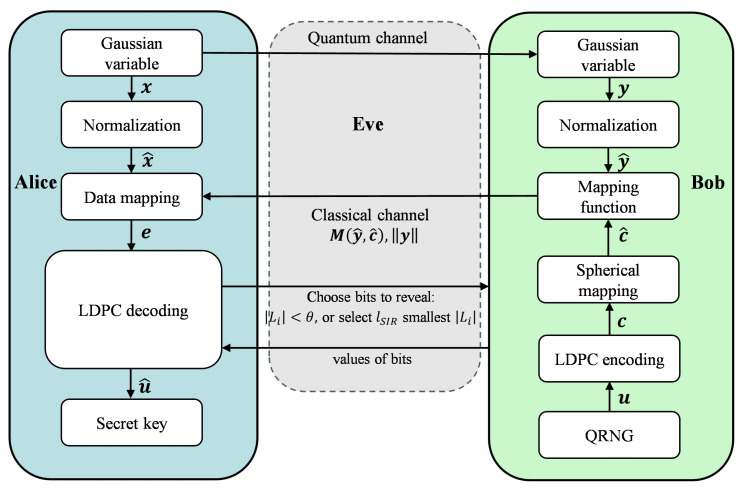
Schematic diagram of the proposed protocols.

In this section, we introduce two rate-adaptive IR protocols for CV-QKD systems, both of which are designed based on the principle of revealing bits with lower reliability. The schematic diagram of the protocols is shown in Figure 2.

### 3.1. Threshold-Based IR (TIR)

#### 3.1.1. Threshold Selection

**Figure 3:**
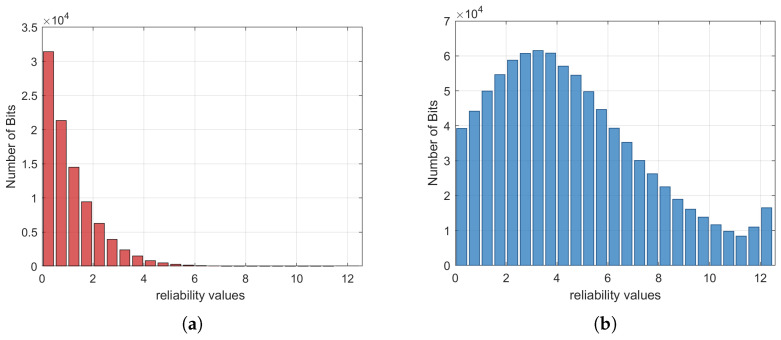
Histogram of decoded bits after decoding failure. (**a**) Correct bits. (**b**) Erroneous bits. The code length is 1,000,000.

The TIR protocol involves setting a threshold θ for the reliability of the bits during the iterative decoding process. Here, define $L_{i}$ as the LLR of the *i*-th bit and define the reliability of the *i*-th bit as the absolute value of $L_{i}$, denoted as $|L_{i}|$. The determination of the threshold θ is precisely based on the reliability value $|L_{i}|$ difference between erroneous bits and correct bits. To clarify how this difference supports the selection of θ, we verify this through a practical scenario: take a rate 0.02 LDPC code with SNR = −15.1 dB and analyze one hundred failed codewords. Histograms of reliability values $L_{i}$ for correct bits and erroneous bits after decoding failure are drawn in Figure 3a and Figure 3b, respectively. It is shown that the reliability value $|L_{i}|$ of erroneous bits is obviously smaller than that of most correct bits, which can be used to distinguish them efficiently. Hence, for a failed codeword, one can set a suitable threshold θ. If the reliability value $\left|L_{i}\right|<\theta$, the corresponding bits are classified into the set of suspicious bits, which have a high probability of being erroneous bits and are selected to be revealed.

#### 3.1.2. TIR Protocol

In this paper, we adopt multidimensional reverse reconciliation. Alice and Bob both have correlated Gaussian sequences, x and y, which satisfy $x∼N{\left(0,\sigma_{x}^{2}\right)}^{d}$ on Euclidean space $R^{d}$ and y=x+z, where $z∼N{\left(0,\sigma_{z}^{2}\right)}^{d}$ and *d* indicates the multidimensional IR dimension. Subsequently, they normalize x and y into $\hat{x}$ and $\hat{y}$, where $\hat{x}=x/\left||x|\right|$ and $\hat{y}=y/\left||y|\right|$. Here ||·|| denotes a norm of a vector. The uniform distribution of the sequences $\hat{x}$ and $\hat{y}$ is satisfied by their distribution on the surface of the unit sphere $S^{d-1}$, which centers on zero.

Using the QRNG, Bob generates a binary sequence u. He then employs LDPC encoding to produce the codeword c. However, in order to adapt c for multidimensional reconciliation, c must be converted into a binary spherical sequence $\hat{c}$ by

(3) $$\hat{c}=\left(\hat{c_{1}},\hat{c_{2}},\ldots ,\hat{c_{d}}\right)\to \left(\frac{{\left(-1\right)}^{c_{1}}}{\sqrt{d}},\frac{{\left(-1\right)}^{c_{2}}}{\sqrt{d}},\ldots ,\frac{{\left(-1\right)}^{c_{d}}}{\sqrt{d}}\right).$$

After acquiring $\hat{y}$ and $\hat{c}$, Bob uses $\hat{y}$ and $\hat{c}$ to compute $M(\hat{y},\hat{c})$, also known as the mapping function. This mapping function satisfies the following condition: $M\left(\hat{y},\hat{c}\right)\cdot \hat{y}=\hat{c}$. Bob transmits $M(\hat{y},\hat{c})$ to Alice through the classical channel. Then, Alice transforms $\hat{x}$ into e using the mapping function $e=M\left(\hat{y},\hat{c}\right)\cdot \hat{x}$.

Alice uses the parity-check matrices of the LDPC code and sum-product algorithm to recover sequence e into sequence $\hat{u}$. During the iterative decoding process, while calculating the LLR, Alice sets a threshold θ for $\left|L_{i}\right|$. She identifies the set of indices $I=\{i||L_{i}|<\theta \}$ and sends I to Bob.

Bob determines the values of the bits in I and returns the values to Alice. If Bob confirms that the *i*-th bit is 1, Alice sets $L_{i}$ to a large negative value, thereby forcing the bit to be interpreted as 1 in subsequent iterations. Conversely, if Bob confirms that the *i*-th bit is 0, Alice sets $L_{i}$ to a large positive value, thereby forcing the bit to be interpreted as 0 in subsequent iterations. Alice continues the iterative decoding with the modified LLRs. If the decoding process is successful, Alice and Bob will possess symmetric secret keys, which are retained for final secret keys; if decoding fails, the secret keys are discarded outright to ensure the absolute security of the final secret keys.

Finally, privacy amplification decreases the information leakage that occurs through the preceding quantum and raw key generation processes. The final secret keys can thus be established by Alice and Bob using a robust randomness extraction protocol.

### 3.2. Sorting-Based IR (SIR)

The SIR protocol for revealing bits with lower reliability involves sorting the $\left|L_{i}\right|$ during the iterative decoding process and selecting a certain number of bits with lower reliability for disclosure. This protocol effectively enables adaptive reconciliation. Before IR, Alice and Bob calculate the optimal code rate $R_{\mathrm{SIR}}$ based on the time-varying SNR of the quantum channel. Then, they select a high-performance original LDPC code whose code rate is close to $R_{\mathrm{SIR}}$. Typically, the original LDPC code rate is defined as

(4) $$R_{0}=\frac{n-m}{n}.$$

Here, *m* and *n* correspond to the number of rows and columns of the LDPC parity-check matrix H, respectively. Subsequently, to achieve the target reconciliation efficiency, it is necessary to reveal $l_{\mathrm{SIR}}$ bits with lower reliability so that the original code rate equals the optimal code rate. This relationship is formulated as

(5) $$R_{\mathrm{SIR}}=\frac{(n-l_{\mathrm{SIR}})-m}{n-l_{\mathrm{SIR}}}=\frac{n-m-l_{\mathrm{SIR}}}{n-l_{\mathrm{SIR}}}.$$

In the SIR protocol, the main differences from the TIR protocol are as follows: Alice uses LDPC decoding to recover sequence e into sequence $\hat{u}$. During the iterative decoding process, while calculating the LLR, Alice takes the $\left|L_{i}\right|$ and sorts them in ascending order. Then, Alice identifies the indices $i_{1},i_{2},\ldots ,i_{l_{\mathrm{SIR}}}$ corresponding to the $l_{\mathrm{SIR}}$ smallest values, such that $|L_{1}\left|\;\leq \;\right|L_{2}\left|\;\leq \ldots \leq \;\right|L_{l_{\mathrm{SIR}}}|$. She constructs the index set $I=\{i_{1},i_{2},\ldots ,i_{l_{\mathrm{SIR}}}\}$ and sends I to Bob.

## 4. Analysis of the TIR and SIR Protocols

In this section, we perform an analysis of the TIR and SIR protocols, focusing on their code rate, reconciliation efficiency, and complexity.

### 4.1. Code Rate

In CV-QKD, we need rate-adaptive and high-efficiency codes since errors must be efficiently corrected at low SNRs to increase the SKR and the secure transmission distance. The TIR protocol identifies bits with lower reliability using the threshold θ: bits with reliability $|L_{i}|\;<\theta$ are selected for revelation. Thus, the code rate is calculated as

(6) $$R_{\mathrm{TIR}}=\frac{(n-l_{\mathrm{TIR}})-m}{n-l_{\mathrm{TIR}}}=\frac{n-m-l_{\mathrm{TIR}}}{n-l_{\mathrm{TIR}}},$$

where $l_{\mathrm{TIR}}$ denotes the number of revealed bits and depends on both the threshold θ and the channel SNR. This results in a passive rate adjustment: $l_{\mathrm{TIR}}$ varies with SNRs, since more bits have $|L_{i}|\;<\theta$ at low SNRs, which increases $l_{\mathrm{TIR}}$ and lowers $R_{\mathrm{TIR}}$. However, in the SIR protocol, rate-adaptive adjustment of the code rate is achieved by adjusting the number of disclosed bits with lower reliability according to variations in SNRs. Specifically, it first sorts the bits in descending order of their reliability $|L_{i}|$ and then calculates the optimal code rate $R_{\mathrm{SIR}}$, which is given by $R_{\mathrm{SIR}}=\beta \cdot C\left(s\right)$ using Equation (2). Finally, to ensure that the original code rate $R_{0}$ is equal to $R_{\mathrm{SIR}}$, we reveal $l_{\mathrm{SIR}}$ bits with lower reliability. According to Equation (5), the $l_{\mathrm{SIR}}$ is calculated as

(7) $$l_{\mathrm{SIR}}=\frac{\left(n-m\right)-R_{\mathrm{SIR}}\cdot n}{1-R_{\mathrm{SIR}}}.$$

Algorithm 1 demonstrates the code rate calculation process of the SIR protocol. In summary, both the TIR and SIR protocols proposed in this paper can achieve adaptive adjustment of the code rate.
**Algorithm 1** The code rate calculation process of the SIR protocol.**Step 1** Obtain the channel capacity C(s) based on the practical SNR.**Step 2** Determine the target reconciliation efficiency β.**Step 3** Sort the bits in descending order of reliability $|L_{i}|$.**Step 4** Obtain the optimal code rate $R_{SIR}$ using Equation (2).**Step 5** Determine the $l_{SIR}$ to disclose $R_{0}\to R_{SIR}$ using Equation (7).

### 4.2. Reconciliation Efficiency

A higher reconciliation efficiency is required at low SNRs in order to meet the criterion that the SKR is greater than zero. From Equation (2), we know that the code rate is the major factor affecting reconciliation efficiency. Thus, in the TIR protocol, the reconciliation efficiency is defined by Equations (2) and (6) as

(8) $$\beta_{\mathrm{TIR}}=\frac{R_{\mathrm{TIR}}}{C(s)}=\frac{n-m-l_{\mathrm{TIR}}}{\left(n-l_{\mathrm{TIR}}\right)\cdot C\left(s\right)}.$$

Additionally, it can also be derived from Equation (2) that under various SNRs, fixed-rate error-correcting codes are unable to sustain high reconciliation efficiency. However, the SIR protocol adjusts the code rate according to variations in SNRs to achieve high reconciliation efficiency across a wide range of SNRs. To be specific, we first set a target reconciliation efficiency, denoted as $\beta_{\mathrm{target}}$. Then, we determine the optimal code rate $R_{\mathrm{SIR}}$ under a given SNR based on Equation (2). Finally, we calculate the number of bits with lower reliability to be disclosed using Equation (7), such that the original code rate $R_{0}$ is adjusted to the optimal code rate $R_{\mathrm{SIR}}$, thereby achieving a high reconciliation efficiency.

### 4.3. Complexity

We conduct an analysis of the time complexity and space complexity for the TIR and SIR protocols. The time complexity O(f(α)) and space complexity O(g(α)) are defined as the asymptotic time cost and storage cost of an algorithm, respectively, both of which are associated with the algorithm’s scale, and specifically described using the O-notation [30]. In this context, f(α) and g(α) are functions that characterize the growth rates of running time of the algorithm and storage demand, with both dependent on the input size α. According to the notations above, the time complexity of the TIR protocol is O(n), since it only requires comparator operations to determine if satisfies $|L_{i}|\;<\theta$. The computational cost thus grows linearly with the block length *n*, and the dominant factor is the number of decoding iterations. In comparison, the SIR protocol requires an additional sorting of the reliability. A direct sorting implementation has complexity O(nlogn), which increases with the block length more rapidly than the TIR protocol. In terms of space complexity, both protocols scale linearly with the block length. For the TIR protocol, the memory requirement is minimal, involving only the storage of indices of revealed bits. The SIR protocol requires additional buffers to store sorted indices and to compute the number of disclosed bits, which leads to slightly higher memory usage but still of linear order. In addition, we analyzed the complexity of other rate-adaptive reconciliation protocols, including the Raptor and MDA protocols. Raptor codes consist of an outer pre-code and an inner Luby transform (LT) code. Therefore, the two-stage architecture also presents challenges. For the Raptor protocol, the complexity of precoding increases dramatically with block length, making it unsuitable for practical applications at low SNRs [8]. Furthermore, LT code decoding requires dynamic symbol generation and confidence propagation, resulting in a complexity of O(nlogn), exceeding that of LDPC-based protocols. The MDA protocol, while sharing the O(n) complexity of the TIR protocol for random bit revelation, may require multiple decoding attempts, which multiplies the base complexity. Overall, both the TIR and SIR protocols maintain a linear order of computational and storage complexity, providing positive implications for the practical implementation of CV-QKD systems.

## 5. Simulation Results

In this section, we study the performance of the proposed TIR and SIR protocols, focusing on reconciliation efficiency, FER, and the SKR. We employ MET-LDPC codes in our simulations. For MET-LDPC codes, different types of edges have independent degree distribution functions. Here, the degree refers to the number of edges connected to variable nodes or check nodes. The detailed degree distribution we use in this paper is shown in Table 1, where ν(r,x) denotes the generating polynomial for the variable node types and μ(x) denotes the generating polynomial for the check node types. For more information about the degree distribution of MET-LDPC codes, refer to [9]. The dimension *d* of multidimensional reconciliation is set to 8 in the simulation that follows.

**Figure 4:**
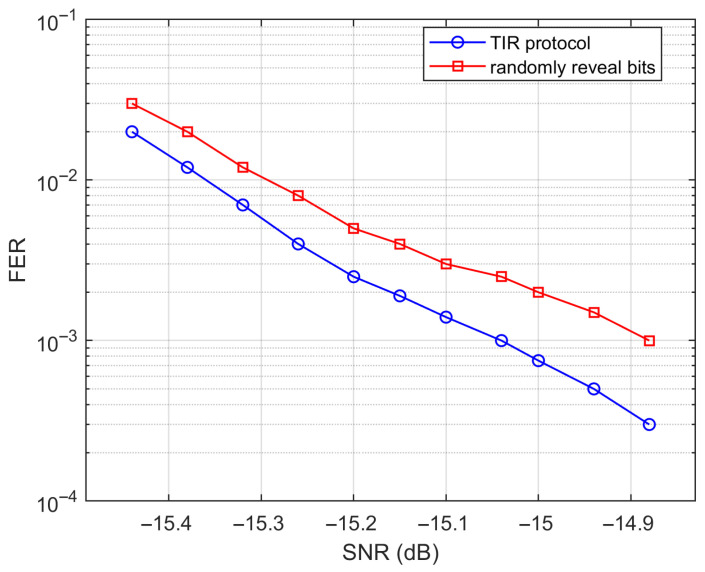
FER comparison between the TIR protocol and random bit revelation.

Figure 4 illustrates the FER performance comparison between the TIR protocol with threshold-based bit revelation and a baseline strategy that randomly reveals the same number of bits, where the 0.02 MET-LDPC code, as presented in Table 1, is used in the comparison. As observed, the method of setting the threshold θ to reveal bits consistently outperforms random revelation in the SNR range from −15.4 to −14.9 dB. This demonstrates that selectively revealing bits with $\left|L_{i}\right|<\theta$ significantly enhances decoding performance and reduces the FER at low SNRs.

**Figure 5:**
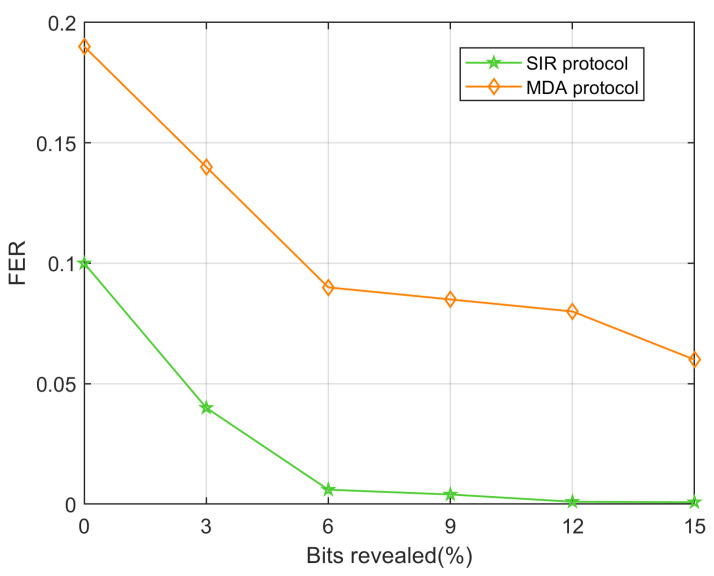
FER comparison under varying bit revelation ratios.

Figure 5 shows the FER performance of the SIR protocol compared with the MDA protocol [25] under varying bit revelation ratios at SNR = −15.23 dB. In the proposed SIR protocol, bits are sorted according to $\left|L_{i}\right|$, and the least reliable ones are selectively revealed. In contrast, the MDA protocol randomly reveals bits for LLR adjustment after decoding failure. As shown in the Figure 5, the proposed SIR protocol consistently achieves a lower FER across all tested revelation ratios. Specifically, when the bit revelation ratio exceeds 6%, the FER of the SIR protocol drops below 0.01, while the MDA protocol still maintains a relatively high FER of around 0.08. This clearly demonstrates that the proposed protocol, which reveals bits with lower reliability, outperforms the MDA protocol [25] with random bit disclosure.

**Figure 6:**
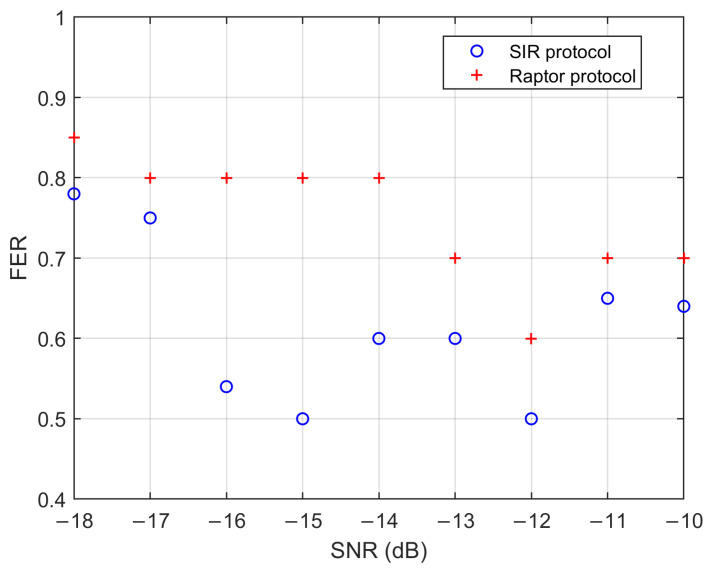
Comparison of FER performance between the SIR protocol and Raptor protocol at low SNRs.

To further study the performance of our proposed SIR protocol at low SNRs, we compare the FER with the Raptor protocol in reference [6], as shown in Figure 6. The target reconciliation efficiency is set to 96% in the SNR range of −18 and −13 dB, and 95% of −12 to −10 dB. As shown in Table 2, we evaluate the number of revealed bits $l_{\mathrm{SIR}}$ to convert the original code rate $R_{0}=$ 0.02, 0.05, 0.1 into the optimal code rate $R_{\mathrm{SIR}}$ under various SNR conditions, in order to maintain the target reconciliation efficiency. Although Figure 6 shows the FER is relatively high, this is a reasonable trade-off to achieve high reconciliation efficiency at low SNRs. Therefore, the simulation results demonstrate that all points (FER < 1) have the desired reconciliation efficiency. In the SNR range between −18 and −10 dB, the SIR protocol has a smaller FER, indicating a greater SKR.

**Figure 7:**
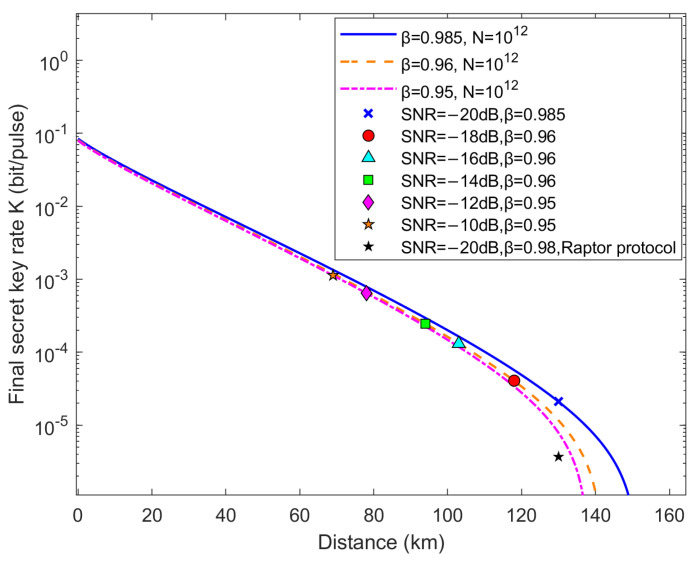
Finite-size secret key rate vs. distance. The cross points, circle points, triangle points, block points, rhombus points, and orange five-pointed stars correspond to our simulation results. The black five-pointed stars are derived from simulations conducted in accordance with the Raptor protocol in reference [6], with an FER of reconciliation of 0.99. The asymptotic theoretical SKRs under different SNRs are represented by a solid line, dashed line, and dot-dash line, respectively. Alice’s modulation variance $V_{A}$ has been optimized, and the other parameters configured are listed as follows: excess noise ξ=0.01, detection efficiency η=0.6, quantum channel attenuation factor α=0.2 dB/km, and electronic noise $v_{el}=0.015$.

Figure 7 presents the finite-size SKR results of the SIR protocol for a CV-QKD system with one-way reverse reconciliation. In practice, the modulation variance $V_{A}$ is applied to approximate the theoretical ideal value by adjusting it in real time based on various channel characteristics. Nevertheless, it is not feasible to obtain simulation results covering the full SNR range from −20 to −10 dB. Thus, we simulate the reconciliation efficiency at low SNRs and present the simulation in Figure 7. Specifically, Figure 7 shows the finite-size SKR results for a block length of $N=10^{12}$ at SNRs of −20, −18, −16, −14, −12, and −10 dB, respectively, while also providing the corresponding asymptotic theoretical SKR. The SKR of our adaptive SIR protocol is calculated using Equation (1), and other parameters remain consistent with those in reference [6]. Given that some bits are used for parameter estimation, we designate half of the bits for parameter estimation and the other half for symmetric secret key extraction (n=N/2) in IR. The simulation results indicate that when the FER of reconciliation is 0.99, the SIR protocol can achieve a reconciliation efficiency of 98.5%, with a theoretical transmission distance exceeding 165 km.

## 6. Conclusions

In this paper, we proposed two rate-adaptive IR protocols for CV-QKD systems, which dynamically adjusted the code rate and improved reconciliation efficiency at low SNRs. The TIR protocol disclosed bits with $\left|L_{i}\right|<\theta$, while the SIR protocol revealed a fixed number of the least reliable bits, allowing dynamic adjustment of the code rate to the time-varying nature of the quantum channel of CV-QKD systems. The analysis demonstrated the overall effectiveness of the proposed TIR and SIR protocols in terms of key metrics including code rate, reconciliation efficiency, and complexity. The simulation results showed that selectively revealing bits with lower reliability outperformed randomly revealing bits. Moreover, the SIR protocol reduced the FER and improved reconciliation efficiency, achieving high reconciliation efficiency over a wide range of SNRs and even attaining over 98.5% efficiency when the SNR dropped below −20 dB. Furthermore, the relationship between the finite-size SKR and transmission distance confirmed the superiority of our scheme. Our work may contribute to the development of practical applications for CV-QKD systems. 

## Figures and Tables

**Table 1:** The degree distribution of MET-LDPC codes.

Code Rate	Degree Distribution
0.1	$\nu \left(r,x\right)=0.0775r_{1}x_{1}^{20}x_{2}^{20}+0.0475r_{1}x_{1}^{3}x_{2}^{22}+0.875r_{1}x^{3}$
	$\mu \left(x\right)=0.0025x_{1}^{11}+0.0225x_{1}^{12}+0.03x_{2}^{2}x_{3}+0.845x_{2}^{3}x_{3}$
0.05	$\nu \left(r,x\right)=0.04r_{1}x_{1}^{21}x_{2}^{34}+0.03r_{1}x_{1}^{3}x_{2}^{34}+0.93r_{1}x^{3}$
	$\mu \left(x\right)=0.01x_{1}^{8}+0.01x_{1}^{9}+0.41x_{2}^{2}x_{3}+0.52x_{2}^{3}x_{3}$
0.02	$\nu \left(r,x\right)=0.0225r_{1}x_{1}^{2}x_{2}^{57}+0.0175r_{1}x_{1}^{3}x_{2}^{57}+0.96r_{1}x_{3}$
	$\mu \left(x\right)=0.010625x_{1}^{3}+0.009375x_{1}^{7}+0.6x_{2}^{2}x_{3}+0.36x_{2}^{3}x_{3}$

**Table 2:** The detailed results of the SIR protocol.

$R_{0}$	SNR (dB)	$l_{\mathrm{SIR}}$	$R_{\mathrm{SIR}}$
0.02	−18	9210	0.01089
	−17	6408	0.01368
	−16	2869	0.01718
0.05	−15	29,069	0.02156
	−14	23,605	0.02703
	−13	16,700	0.03387
	−12	8421	0.04193
0.1	−11	1005	0.05238
	−10	742	0.06531

## Data Availability

The original contributions presented in this study are included in the article. Further inquiries can be directed to the corresponding author(s).
